# *Vibrio cholerae* Biofilms and Cholera Pathogenesis

**DOI:** 10.1371/journal.pntd.0004330

**Published:** 2016-02-04

**Authors:** Anisia J. Silva, Jorge A. Benitez

**Affiliations:** Morehouse School of Medicine, Department of Microbiology, Biochemistry and Immunology, Atlanta, Georgia, United States of America; Oxford University Clinical Research Unit, VIETNAM

## Abstract

*Vibrio cholerae* can switch between motile and biofilm lifestyles. The last decades have been marked by a remarkable increase in our knowledge of the structure, regulation, and function of biofilms formed under laboratory conditions. Evidence has grown suggesting that *V*. *cholerae* can form biofilm-like aggregates during infection that could play a critical role in pathogenesis and disease transmission. However, the structure and regulation of biofilms formed during infection, as well as their role in intestinal colonization and virulence, remains poorly understood. Here, we review (i) the evidence for biofilm formation during infection, (ii) the coordinate regulation of biofilm and virulence gene expression, and (iii) the host signals that favor *V*. *cholerae* transitions between alternative lifestyles during intestinal colonization, and (iv) we discuss a model for the role of *V*. *cholerae* biofilms in pathogenicity.

## Introduction

The water-borne diarrheal disease cholera is caused by the gram-negative and motile bacterium *Vibrio cholerae* of serogroup O1 and O139. *V*. *cholerae*, as other members of the *Vibrionaceae* family, are common inhabitants of aquatic ecosystems. In regions where cholera is endemic, occurrence of the disease follows a seasonal pattern that correlates with climatic changes [1–8]. Import of *V*. *cholerae* O1 into nonendemic areas with poor sanitation commonly results in rapid dissemination of the disease through a fast fecal–oral route that takes advantage of the transient hyperinfective stage of *V*. *cholerae* present in fresh cholera stool [9–12]. *V*. *cholerae* O1 can be divided into two biotypes, classical and El Tor, which differ in the severity of clinical symptoms and the expression and regulation of major virulence factors [13]. Humans have experienced seven cholera pandemics. The seventh and current pandemic is characterized by the predominance of the O1 serogroup of the El Tor biotype, with periodic emergence of serogroup O139, which originated from the El Tor biotype and exhibits a new lipopolysaccharide (LPS) and a capsule [14].

## Virulence Factors

The two major virulence factors expressed by *V*. *cholerae* O1 and O139 are (i) cholera toxin (CT), an AB_5_ family ADP-ribosyltransferase responsible for the profuse rice-watery diarrhea typical of this disease [13], and (ii) the toxin-coregulated pilus (TCP), a type IV pilus that mediates adherence and microcolony formation and is required for intestinal colonization in neonate mice and humans [15–17]. The genes encoding the CT subunits *ctxA* and *ctxB* constitute an operon within the prophage form of the filamentous phage CTXΦ [18]. The genes required for TCP biogenesis form a large cluster known as the *V*. *cholerae* pathogenicity island (VPI) or TCP island [19]. Within this cluster, *tcpA* encodes the major pilus subunit.

Also important for the pathogenicity of the cholera bacterium is the expression of a sheathed polar flagellum driven by sodium motive force (SMF) [20]. Flagellar motility is a complex phenotype that requires (i) the synthesis, export, and assembly of the flagellum and its motor; (ii) conversion of SMF to flagellum rotation work; and (iii) control of the direction of flagellum rotation by chemotaxis. The expression of motility requires a hierarchical regulatory cascade that involves the alternative RNA polymerase subunits σ^54^ and σ^28^ and the σ^54^-dependent transcriptional activators FlrA and FlrC [21]. In addition, evidence has grown suggesting that flagellar motility participates in the regulation of virulence gene expression. For instance, mutations or chemical inhibitors that result in a paralyzed flagellum enhance the transcription of *ctxA* and *tcpA* [22–26]. The mechanism by which cessation of motility enhances virulence gene expression is unknown.

## Stress Response

*V*. *cholerae* has evolved to effectively colonize disparate ecological niches: the nutrient-rich human small intestine and aquatic environments. In the aquatic environment, *Vibrios* must withstand diverse physical, chemical, and biological stresses that include nutrient limitation, extreme temperatures, oxidative stress, bacteriophage predation, and protozoan grazing [27,28]. In the gastrointestinal tract, *Vibrios* are exposed to low pH, bile acids, elevated osmolarity, iron limitation, antimicrobial peptides, and intermittent nutrient deprivation [29]. Thus, both environments pose common and specific challenges to bacterial growth and multiplication. The human small intestine, nevertheless, provides a superior bounty of nutrients compared to aquatic environments. Consistently, *V*. *cholerae* can grow to high titers in the human gut, and cholera patients can shed 10^7^–10^9^ virulent *Vibrios* per mL in the rice-watery stool [12]. In order to reach high titers in the gut, *V*. *cholerae* must overcome as many stressful conditions as it requires to survive and persist outside the human host. Proof of this is that disruption of genes encoding the general stress response regulator RpoS (σ^S^) or the RNA polymerase σ^E^ subunit (RpoE) that mediates the envelope stress response results in significant attenuation of *V*. *cholerae* virulence and its capacity to colonize the small intestine [30,31]. Thus, whether in the human host or in the aquatic environment, the cholera bacterium employs common survival strategies. These stratagems involve (i) the activation of general and specific stress responses, (ii) expression of flagellar motility and chemotaxis, (iii) attachment to surfaces, (iv) development of multicellular sessile communities, and (v) detachment. Particularly critical to *V*. *cholerae* survival in the host and estuarine waters is its ability to switch between motile (planktonic) and sessile (biofilm) lifestyles in response to chemical and physical changes in the extracellular milieu.

## *V*. *cholerae* Biofilms

Biofilms are microbially derived sessile communities characterized by cells that are attached to a substratum, an interface, or to each other; are embedded in a self-produced matrix; and exhibit an altered phenotype with respect to growth rate and transcription profile [32,33]. This definition includes communities of *Vibrios* anchored to abiotic surfaces or to biotic substrata such as the human intestinal mucosa or the chitinous exoskeleton of crustaceans, *Vibrio* aggregates in suspension, floccules, and pellicles formed at the liquid–air interface of static cultures.

It has been established that *V*. *cholerae* cells in planktonic, monolayer, and mature biofilm stages differ in their global transcription profile [34,35]. A major event in the transition from planktonic to biofilm lifestyle is the down-regulation of motility gene expression and induction of genes required for the biosynthesis of the biofilm extracellular matrix [34,35]. We note that the signaling pathway by which an initial attachment to a surface induces significant changes in the transcriptome is unknown. In the mature biofilm microenvironment, cells are packed within a smaller volume, and nutrient accessibility and the elimination of toxic metabolic products is limited by diffusion. These conditions favor an early entry of cells into quorum sensing mode and stationary phase. As an example, the cholera autoinducer 1 was shown to accumulate to a higher concentration in biofilms compared to planktonic cells, resulting in earlier expression of the quorum sensing regulator HapR [36]. In turn, HapR enhances the expression of the stationary phase sigma factor RpoS [37]. Thus, the mature biofilm exhibits a gene expression pattern that favors resistance to environmental stressors.

The regulation and structure of biofilms formed under laboratory conditions has been the subject of much study and several reviews [38–40]. It is well established that the intracellular concentration of the second messenger cyclic diguanylic acid (c-di-GMP) controls the transition between *V*. *cholerae* planktonic and biofilm lifestyles [41–46]. c-di-GMP is synthesized from GTP by the activity of diguanylate cyclase (DGC) exhibiting GGDEF domains and degraded to GMP by phosphodiesterases (PDE) exhibiting EAL or HD-GYP domains [47]. The *V*. *cholerae* genome encodes 31 GGDEF, 22 EAL, 9 HD-GYP, and 10 combined GGDEF-EAL domain proteins [48]. Most of these proteins display a modular architecture with added sensor, effector, and DNA binding domains.

Three major regulators sense the intracellular concentration of c-di-GMP: the σ^54^-dependent activator FlrA required for the expression of flagellar motility [21,49] and the biofilm activators VpsR [50] and VpsT [51,52]. Five membrane-bound DGC (CdgA, H, L, K, and M) act additively to increase the c-di-GMP pool and promote dimerization and activation of VpsT to induce biofilm formation [46]. The *V*. *cholerae* genome also encodes five proteins containing PilZ domains, a separate family of c-di-GMP binding proteins [53]. The role of PilZ domain proteins in regulating motility, biofilm, and virulence is unclear. Deletion of three genes encoding PilZ domain proteins resulted in reduced motility, diminished biofilm formation, and intestinal colonization [53]. The negative effect of these deletions on motility and biofilm formation is unexpected, given that these cellular processes are inversely regulated by c-di-GMP. It is possible that the deleted PilZ proteins affect motility and colonization by a mechanism unrelated to c-di-GMP.

The genes (*vps*) responsible for making the *V*. *cholerae* exopolysaccharide matrix are located in two clusters (*vpsU*, *vpsA-K*) and *vpsL-Q* on *V*. *cholerae* chromosome I [54]. These clusters comprise two operons in which *vpsA* and *vpsL* are the first genes of operon I and II, respectively [54]. A third gene cluster, *rbmA-F*, located between the *vpsA-K* and *vpsL-Q* operons and *bap*1 encode protein components of the biofilm matrix [55]. RbmA is only expressed on the surface of cells that make the exopolysaccharide and functions to enhance cell-to-cell adhesion [56]. In addition, RbmA can undergo limited proteolysis to a form capable of interacting with cells not expressing *vps*, thereby recruiting planktonic cells to the growing biofilm [57]. Bap1 promotes adherence of the developing biofilm to a surface [56,58,59], and RbmC cooperates with Bap1 in the formation of flexible envelopes that grow as cells divide and stabilize the biofilm [56]. Deletion of *rbmA*, *rbmC*, and *bap*1 results in diminished biofilm formation in vitro [55].

The expression of genes in the *vps* and *rbm* clusters is under positive transcription regulation by VpsR and VpsT [40]. When the intracellular concentration of c-di-GMP is high, allosteric activation of VpsR and VpsT enhances the expression of genes required to make the biofilm matrix [60]. In parallel, c-di-GMP binds to FlrA to inhibit its activity and diminish flagellar gene expression [49].

In addition to exopolysaccharide and proteins, the biofilm matrix contains extracellular DNA (eDNA). The eDNA has been suggested to contribute to the structural stability of biofilms, though its precise interaction with other components of the matrix is unclear [61]. In *V*. *cholerae*, the level of eDNA is regulated by nucleases *dns* and *xds*, the latter gene a member of the Pho regulon [62]. A double mutant lacking both nucleases produced enhanced biofilm as a consequence of reduced detachment [63]. It has also been suggested that eDNA can serve as a source of phosphate to biofilm cells [64]. Thus, phosphate starvation could function as a signal for eDNA degradation and biofilm dispersion. This interpretation is consistent with results showing that phosphate limitation negatively affects biofilm formation [65,66]. We note, however, that degradation of eDNA could potentially increase the concentration of cytidine in the biofilm, an allosteric inhibitor of the LacI-family regulator CytR reported to repress *vps* expression and biofilm formation [67]. Hence, much remains to be learned on the role of eDNA, phosphate regulation, and nucleoside catabolism in biofilm formation and dispersal.

## Evidence for Biofilm Formation during Infection

### Microscopic observation of in vivo-formed biofilm-like aggregates

Early microscopic examination of *V*. *cholerae* in association with the intestinal mucosa of adult and infant rabbits revealed patches of *Vibrios* adherent to the mucous coat and along the villi [68–71]. More recently, the use of confocal and intravital two-photon microscopy confirmed the localization of infecting *Vibrios* in the form of clonal microcolonies attached along the villous axis and crypts [72]. Further, examination of human fresh cholera stool reveals the presence of *V*. *cholerae* in the form of both planktonic cells and biofilm-like structures consisting of large clumps of cells [73,74]. However, the composition and architecture of *V*. *cholerae* microcolonies and biofilm-like aggregates formed in vivo has not been established.

### Common genetic determinants of biofilm development and intestinal colonization

There is plentiful evidence suggesting that the capacity of *V*. *cholerae* to develop biofilms is critical to intestinal colonization. The genes required for the adoption of both *V*. *cholerae* lifestyles are expressed during infection in the rabbit ileal loop model [75]. In fact, some biofilm genes (i.e., *vpsA*, *rbmA*) were expressed at a higher level in vivo compared to LB medium [75]. In a suckling mouse single strain infection colonization assay, planktonic *vps* mutants of *V*. *cholerae* O1 (El Tor), which are defective for biofilm formation in vitro, exhibit diminished recovery from intestinal tissue compared to wild type [76]. Deletion of *rbmA* significantly diminished intestinal colonization, while inactivation of *rbmC* and/or *bap*1 had no effect [76]. This finding suggests that biofilms formed during infection may consist primarily of cell aggregates that do not progress beyond the RbmA-dependent clustering stage or that other factors can functionally replace Bap1 and RbmC for biofilms formed in vivo. The above data contradicted an earlier competitive colonization study suggesting that *vps* expression is not required for intestinal colonization by *V*. *cholerae* O139 [77]. Possible explanations for the conflicting results are (i) an unrecognized role of the O139 LPS in adherence to the intestinal mucosa or (ii) masking of the O139 *vps* defect by *Vibrio* exopolysaccharide (Vps) produced by the wild type co-inoculated strain. Nevertheless, expression of *vps* genes were required for *V*. *cholerae* O139 intestinal colonization in the fruit fly *Drosophila melanogaster*, a novel oral infection model that exhibits cholera toxin-dependent lethality and mimics human infection [78,79].

Inhibition of motility, which diminishes biofilm formation in vitro, also negatively affects colonization of the small intestine [24,77,80]. Nonmotile mutants exhibit reduced attachment and monolayer formation on abiotic surfaces as well as adherence to the intestinal epitlelium in animal models [24,81–83]. The mechanism by which flagellar motility affects surface attachment is not fully understood. Initial studies suggested that adherence could be modulated by sodium flux through the flagellar motor and membrane potential [80,84]. A later study suggested that the flagellum allows *Vibrios* to swim along a surface and act synergistically with pili in a scanning mode for strong surface–pili interactions [85]. The above mechanisms are difficult to dissect, as conditions that slow the flagellum (i.e., tethering the cell to a surface, high viscosity) could diminish sodium flux through the motor and perturb the membrane potential. Likely, both mechanisms can contribute to bacterial adherence. The mannose-sensitive hemaglutinin (MSHA) is important for biofilm development on borosilicate surfaces [81,86] and on the exoskeletons of the planktonic crustacean *Daphnia pulex* [87]. In vitro, the polar flagellum was proposed to act synergistically with the MSHA to promote surface skimming and attachment [85]. However, deletion of *mshA* does not affect intestinal colonization [16,17]. Thus, the flagellum could act in concert with other pili to promote attachment during infection. We suggest that the TCP pilus, which mediates microcolony formation and attachment to polarized Caco-2 cells [88–91], could act synergistically with the flagellum to promote adherence to intestinal cells.

Altogether, the fact that production of Vps, RbmA, and motility are required for efficient colonization of the small intestine suggests that *V*. *cholerae* must be capable of adopting both motile and biofilm lifestyles during the infective process to successfully colonize the small bowel.

### Changes in the *V*. *cholerae* c-di-GMP pool during infection

Fluctuations in the intracellular c-di-GMP pool during infection suggests that *V*. *cholerae* senses environmental cues in the small intestine that favor lifestyle transitions from sessile to motile and vice versa. It was reported that an increase in the c-di-GMP pool represses virulence gene expression [92–94]. These finding led to a model in which *V*. *cholerae* enters the small intestine in a stage characterized by an elevated c-di-GMP pool such as a biofilm, but induction of PDE and/or repression of DGC activities act to lower the c-di-GMP pool to achieve maximal expression of motility and virulence genes [48]. However, recombination-based in vivo expression technology (RIVET) identified genes specifically expressed late in infection encoding GGDEF domain proteins with DGC activity [95]. The authors suggested that *V*. *cholerae* can increase its c-di-GMP pool late in infection, a condition that promotes biofilm development, prior to exiting the host in preparation for life in the aquatic environment. This model does not explain, however, why planktonic *vps* mutants exhibit diminished capacity to establish infection in the suckling mouse model. Recent studies suggest *V*. *cholerae* may occupy distinct niches along the small intestine, requiring location-specific factors [72,96]. We suggest that *Vibrios* must respond to changes in the chemical composition of their surroundings during infection by switching between sessile and motile lifestyles. This ability could provide fitness and explain why both planktonic nonmotile and *vps* mutants exhibit diminished intestinal colonization capacity.

## The Coordinate Regulation of Virulence Gene Expression and Biofilm Development

**Fig 1:**
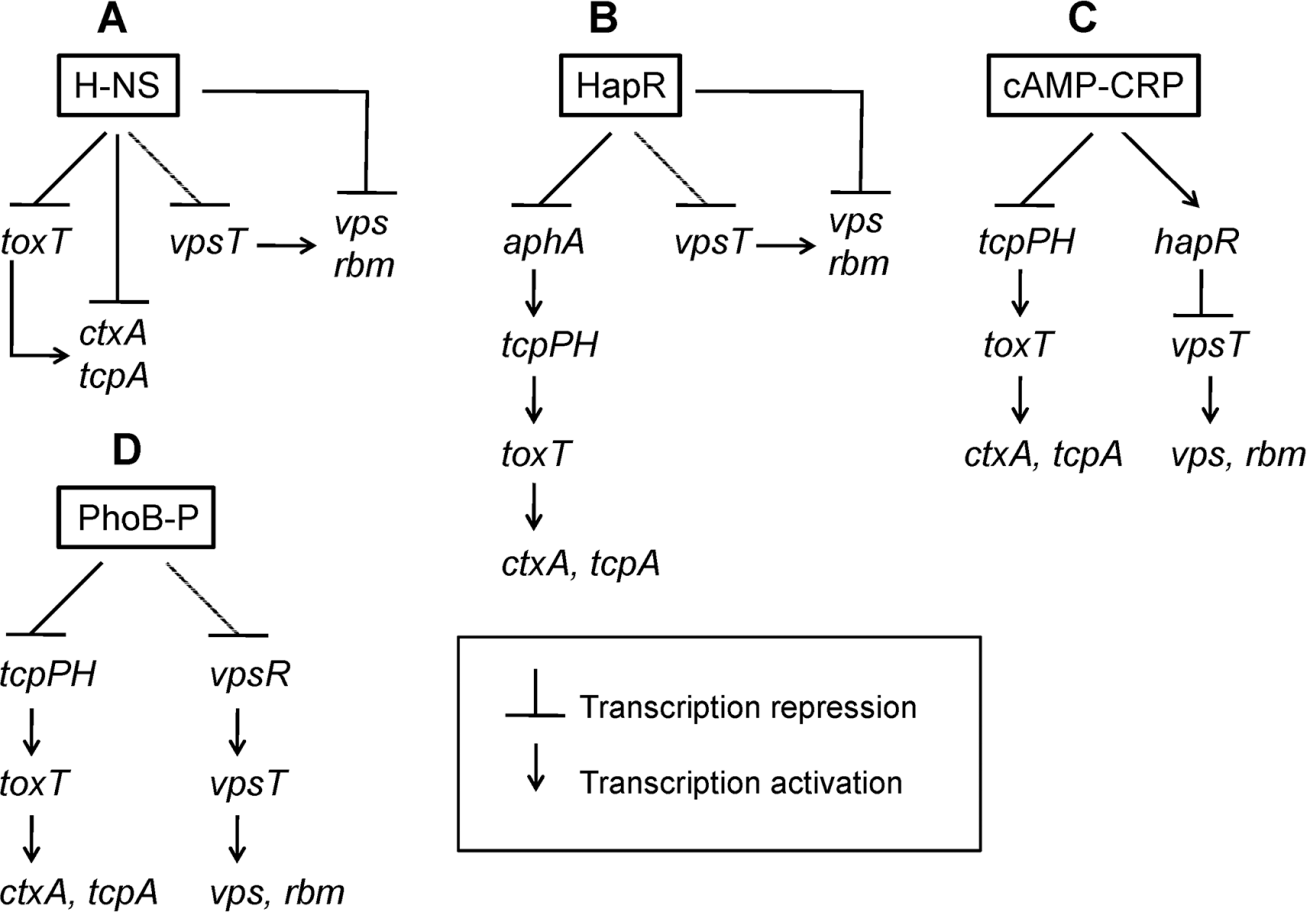
Coordinate regulation of virulence gene expression and biofilm development. (A) The nucleoid organizer H-NS silences the transcription of *ctxA* and *tcpA* directly and via repression of *toxT*. Expression of *ctxA* and *tcpA* is made possible by the action of proteins ToxT and IHF that function as antirepressors. H-NS silences the transcription of *vpsT* and downstream *vps* and *rbm* genes. Activation of biofilm genes is made possible by a VpsR- and VpsT-dependent antirepression cascade. (B) At high cell density, the quorum sensing regulator HapR diminishes *ctxA* and *tcpA* expression by repressing *aphA*. HapR terminates the transcription of *vpsT* to repress biofilm formation. (C) Depending on carbon source type and availability, the cAMP receptor protein (CRP) negatively regulates virulence and biofilm formation by repressing the transcription of *tcpPH* and activating HapR. D. Phosphate limitation triggers phosphorylation of PhoB which diminishes virulence gene expression and biofilm by repressing *tcpPH and vpsR*, respectively.

The regulation of virulence gene expression has been the subject of extensive research and recent reviews [97,98]. Expression of CT and TCP is regulated by a complex regulatory network (Fig 1). At the top of the Tox regulatory cascade, regulators AphA and AphB enhance the transcription of the transmembrane regulators TcpP and TcpH [99,100]. TcpP/H, in concert with transmembrane regulators ToxR/S [101,102], activate the expression of the soluble AraC-family regulator ToxT [103]. Finally, ToxT interacts with the *ctxA* and *tcpA* promoters to activate the production of CT and TCP [103]. The dependence of *ctxAB* and *tcpA* expression on the upstream regulators ToxR/S, TcpP/H, and ToxT was confirmed in vivo using the suckling mouse model and RIVET [104].

Numerous signal transduction pathways simultaneously feed sensory information into the Tox cascade and control the c-di-GMP pool to coordinate the expression of major virulence factors and biofilm development (Fig 1). These regulatory connections point to the conclusion that signals that enhance virulence gene expression and biofilm formation are present in the environment of the small intestine creating the evolutionary pressure to coordinate these cellular processes.

### Transcriptional silencing of virulence and biofilm formation by the histone-like nucleoid structuring protein (H-NS)

H-NS is a highly abundant protein that functions as a nucleoid organizer and a transcriptional silencer at promoters exhibiting AT-rich, highly curved DNA. It preferentially silences the transcription of virulence factors acquired by horizontal gene transfer [105]. In *V*. *cholerae*, transcription of the xenogenic genes *ctxA* and *tcpA* is silenced by H-NS (Fig 1A) [106]. Repression of *ctxA* and *tcpA* is antagonized by ToxT and the integration host factor (IHF) [107–109]. The *vps* and *rbm* genes are also silenced by H-NS and are expressed or reset back to silent depending on environmentally induced fluctuations in the c-di-GMP pool (Fig 1A) [110–114]. We showed that activation of VpsR by c-di-GMP antagonizes H-NS repression at the *vpsT* promoter [112]. Then, expression and allosteric activation of VpsT by c-di-GMP antagonizes H-NS repression at the downstream *vps* and *rbm* promoters [112].

Chromatin immunoprecipitation and parallel DNA sequencing (ChIP-Seq) showed a significant trend for H-NS to cluster at regions of the chromatin involved in the expression of virulence (*ctxAB*, TCP island), surface attachment, and biofilm formation (*vps*, *rbm*) [112,113]. Recent studies have led to the view that H-NS organization of the chromatin and transcriptional silencing are interrelated functions in which gene regulation drives nucleoid organization [115]. Clustering of H-NS at sites of the chromatin encoding virulence factors and genes required to make the biofilm matrix could bring these regions into proximity rendering their coordinate regulation more effective. Further, H-NS clustering at these sites could function to synchronize virulence and biofilm formation in response to environmental conditions that affect DNA superhelical density, such as temperature, pH, osmotic shifts, transitions from aerobiosis to anaerobiosis, and starvation [116]. Thus, the above studies suggest that H-NS coordinates biofilm and virulence gene expression at both the transcription initiation and chromatin organization levels.

### Quorum sensing

Quorum sensing is a cell–cell communication process in bacteria that involves the production, release, and subsequent sensing of signaling molecules termed autoinducers. In *V*. *cholerae*, two autoinducer/sensor systems have been identified. The major system consists of cholera autoinducer 1 (CA-1) and its cognate receptor, CqsS, while a second system consists of autoinducer 2 (AI-2) and its cognate receptor, LuxPQ [117,118]. At low cell density, multiple, redundant small regulatory RNAs (sRNAs or *qrr*) enhance expression of the regulator AphA and destabilize the mRNA encoding the regulator HapR [118,119]. Accumulation of CAI-1 at high cell density results in termination of sRNA transcription, down-regulation of *aphA*, and expression of *hapR* [118,119]. The master quorum sensing regulator HapR lowers virulence gene expression by inhibiting the transcription of *aphA* (Fig 1B) [120]. Quorum sensing negatively controls biofilm formation through the regulators AphA and HapR [118]. At low cell density, AphA enhances the expression of the biofilm activator VpsT [121]. At high cell density, HapR diminishes biofilm formation by lowering the intracellular c-di-GMP pool and repressing *vpsT* (Fig 1B) [122]. Thus, in contrast to other bacterial pathogens, quorum sensing acts in *V*. *cholerae* to repress biofilm formation and virulence gene expression.

### Carbon source and nutrient limitation

Bacteria respond to the availability of sugars in the medium through a phosphoryl transfer cascade known as the phosphoenolpyruvate (PEP) phosphotransferase system (PTS) [123]. In the PTS, sugar transport and phosphorylation occur at the expense of PEP through a phosphoryl cascade involving the pathway-specific proteins enzyme I (EI) and HPr, and sugar-specific enzyme II (EII) complexes [123]. The different EII complexes are characterized by their domains (A, B, C), present either on a single or distinct polypeptide chains. In the *Enterobacteriaceae*, phosphorylated glucose-specific EIIA activates adenylate cyclase to make cAMP, which binds to the cAMP receptor protein (CRP) to induce or repress gene expression [123]. In *V*. *cholerae*, the expression of virulence genes is negatively modulated by CRP, which acts to repress transcription of *tcpPH* [100] and activate the expression of HapR (Fig 1C) [124–126]. A mutant lacking EI of the PTS showed diminished colonization in neonate and germ-free adult mice models, suggesting that individual components of the PTS can exert additional regulation over virulence gene expression [127,128]. Carbon source modulates the expression of VpsR and VpsT by controlling the expression of HapR via CRP [124,126,129] and the levels of phosphorylated intermediates of the PTS where phospho-EI and phospho-HPr act to repress *vps* expression [128,130,131].

### Phosphate limitation

Freshwater and estuarine ecosystems where *V*. *cholerae* can survive and persist outside the human host are limited in phosphate content. Similarly, phosphate is a limiting nutrient in the small intestine [62]. Bacteria respond to phosphate limitation through the PhoR/PhoB two-component regulatory system [132]. Under conditions of phosphate limitation, the histidine kinase PhoR interacts with the phosphate transport system (Pst) to activate PhoB by phosphorylation. Phosphorylated PhoB then modulates the transcription of a set of genes known as the Pho regulon [132]. During infection, phosphorylated PhoB diminishes *ctxA* and *tcpA* expression by binding to the *tcpPH* promoter to repress transcription initiation [133]. Phosphate limitation and PhoB negatively control biofilm formation by lowering the expression of VpsR and modifying the c-di-GMP pool [65,66].

## Host Signals That Coordinate Virulence Gene Expression with Biofilm Development

Numerous physical and chemical cues in the gut (i.e., temperature, pH, oxygen tension, osmolarity, bile salts, antimicrobial peptides) can impact the infective process. It is likely that all these factors, at least indirectly, influence virulence and biofilm formation. Compounds that perturb the cell envelope can generate additional stresses, resulting in elevated expression of RNA polymerase subunits σ^E^ and σ^S^ [134]. Intestinal bile exhibits these properties and has received abundant consideration as a host-specific signal that can potentially modulate *Vibrio* behavior in the gut. Intestinal bile is a complex mixture of bile acids, cholesterol, and unsaturated fatty acids and is subject to numerous chemical transformations in the gastrointestinal tract (i.e., removal of amino acid side chains, oxidation, hydroxylation, and dehydroxylation). Crude bile or sodium cholate was found to enhance biofilm formation in a VpsR-dependent manner [135]. This observation is consistent with the recent finding that a mixture of bile acids increased the intracellular c-di-GMP pool, an effect that was quenched in the presence of bicarbonate [136]. Surprisingly, the individual bile salt taurocholate was found to promote biofilm dispersal rather than formation [137]. These differences may reflect the limited capacity of commercial bile preparations to represent the properties of bile secreted into the intestinal lumen.

Bile also modulates virulence gene expression. Treatment of *V*. *cholerae* with a crude ox bile extract resulted in diminished expression of CT and TCP [138]. Subsequent studies with purified bile components showed that unsaturated fatty acids repress the transcription of *ctxA* and *tcpA* [139]. Unsaturated fatty acids were shown to inhibit ToxT activity [140] and its binding to the *ctxA* and *tcpA* promoters [141]. Contrary to the effect of unsaturated fatty acids, bicarbonate enhanced the activity of ToxT and its binding to its target promoters [142,143]. Bile concentration is elevated in the lumen and low in the vicinity of the villi, while bicarbonate exhibits the opposite gradient. Thus, these molecules could act as a location-specific switch, modulating *V*. *cholerae* behavior during infection. In summary, the above studies suggest an inverse regulatory model in which components of bile present in the intestinal lumen favor biofilm formation by enhancing the c-di-GMP pool and, in parallel, suppress the premature expression of the major virulence factors CT and TCP.

## Colonization of the Small Intestine

The small intestine commences at the pyloroduodenal junction and ends at the ileocaecal junction and comprises, successively, the duodenum, jejunum, and ileum. The mucosal side of the small intestine is composed of absorptive polarized epithelial cells (enterocytes) organized in the form of finger-like projections or villi and mucin-secreting goblet cells covered by a protective mucus barrier. The protective mucus coat consists of a firmly adherent inner layer overlaying the villi and a loosely attached outer layer [144]. The thickness and biophysical properties of the mucus barrier varies along the gastrointestinal tract and is determined by the balance between its secretion rate and its erosion through enzymatic degradation and mechanical shear. The total mucus layer thickness is estimated to be 170–123 μM in the duodenum and jejunum and 480 μM in the ileum [145].

*V*. *cholerae* colonization of the small intestine has been extensively studied using the suckling mouse competitive colonization assay [146], infant rabbits [68,147], and rabbit ileal loops [68]. The use of the above animal models in combination with transposon or signature-tag mutagenesis and RNA-Seq has led to the discovery of multiple colonization factors [148–151]. Further, the suckling mouse colonization assay in combination with RIVET has identified genes that are specifically induced during infection [152–155]. Genes that specifically influence intra-intestinal growth fall into two broad categories: those encoding factors that enhance intestinal colonization (i.e., motility) and those that are stringently required for colonization, such as *tcpA* [15,156]. Activities of TCP that promote intestinal colonization include adherence to intestinal cells [90], microcolony formation [88,89], and secretion of the secondary colonization factor TcpF [157].

Contracting cholera involves the oral ingestion of virulent *Vibrios* capable of expressing TCP and CT in the form of planktonic cells or biofilms. *V*. *cholerae* cells in a biofilm exhibit a lower infective dose and outcompete their planktonic counterparts in the suckling mouse colonization assay [158]. The biofilm advantage is transient and does not require the intact biofilm architecture [158]. *V*. *cholerae* biofilms have been reported to be more resistant to acid inactivation [159]. In addition, biofilm-derived cells could be more effective in competing for limiting nutrients in the small intestine, as suggested by their elevated expression of their phosphate uptake system compared to planktonic cells [160].

Wild type (chemotactic, motile) *V*. *cholerae* preferentially colonizes the middle to distal small intestine [96,161,162]. In contrast, motile but nonchemotactic mutants exhibiting counterclockwise flagellum rotation colonized the entire length of the small intestine, while nonmotile or nonchemotactic mutants showing clockwise flagellum rotation exhibited diminished colonization [24,154,161]. It has been suggested that ingestion of hyperinfective biofilms can represent a natural mode of contracting cholera during outbreaks and a fast track for disease dissemination [12]. However, the fact that both motility and chemotaxis positively influence *V*. *cholerae* colonization capacity indicates that *Vibrios* within an infective biofilm must detach and switch to the planktonic lifestyle to effectively colonize the gut. Consistent with this view, Δ*dns*Δ*xds* nuclease-defective mutants that showed diminished detachment of cells from biofilms in vitro also exhibited reduced intestinal colonization capacity, presumably due to inefficient dispersal of the biofilm upon entering the intestinal lumen [63].

*Vibrios* detached from an incoming biofilm must swim toward the intestinal mucosa and penetrate the protective mucus barrier. Flagellar motility could facilitate bacterial attachment to the protective mucus layer in cooperation with the N-acetylglucosamine-binding protein and colonization factor GbpA reported to mediate bacterial adherence to intestinal mucin [163–166]. Flagellar motility can also contribute to the initial penetration of the mucus gel. This view is suppported by the observation that pretreatment of mice with the mucolytic agent N-acetyl-L-cysteine partially restored colonization capacity to nonmotile mutants [72]. Though in the standard suckling mouse model motility is required for overall intestinal colonization, more refined microscopy techniques showed that *V*. *cholerae* colonization of the proximal and distal small intestine exhibit distinct requirements for motility and chemotaxis [72]. It is intriguing that motility was required for *V*. *cholerae* to reach the crypts of the proximal small intestine, but not the distal region protected by a thicker mucus gel [72]. Thus, the mechanism by which *Vibrios* reach their niche outside the intestinal lumen could involve additional factors. This notion is supported by studies showing that the polar flagellum is a less effective locomotion organelle in a high viscocity medium [167] and tends to break in the viscous mucus gel [166]. Penetration of the mucus barrier could be as well facilitated by flagellum-independent locomotion and/or the activity of mucolytic enzymes. A flagellum-independent surface translocation on semisolid media has been reported in *V*. *cholerae*, which required the production of wild-type LPS [168]. In addition, expression of the the the Zn-dependent metalloprotease hemagglutinin (HA)/protease [169,170] enhanced the penetration of a mucin gel in vitro in a column assay [170]. A recent in vitro study suggested that intestinal mucin represses the expression of *vps* genes [171]. We suggest that this effect could prevent the counterproductive formation of sessile cell aggregates and/or biofilms within the protective mucus layer.

Following penetration of the mucus barrier, *Vibrios* locate along the villous axis and crypts in the form of microcolonies [72]. Microcolony formation is thought to be mediated by TCP [89], which is also an adherence factor [90] and promotes biofilm formation on chitin [172]. A recent study showed that microcolonies observed along the villous axis and the crypts are clonal [72]. This finding contrasts with the mechanism by which TCP promotes the formation of nonclonal aggregates in vitro through pilus–pilus interactions [89]. Thus, TCP could contribute to microcolony formation in vivo by a mechanism distinct from pilus–pilus interaction and/or cooperate with additional factors. We do not know if microcolonies are embedded in an exopolysaccharide matrix similar to the biofilms formed in vitro. Further, while *vps* mutants show diminished colonization [76], it has not been determined if microcolony formation is *vps*-dependent.

*Vibrios* along the villous axis and crypts express CT, which binds to its GM_1_ receptor in the apical membrane of intestinal epithelial cells and is internalized by endocytosis. Based on the inverse relationship between motility and virulence gene expression [24,25], cessation of motility upon bacterial attachment to the villi is expected to enhance CT expression. Toxin delivery at this site in close proximity to its GM_1_ receptor is stimulated by low bile and elevated bicarbonate levels. This spatiotemporal pattern of CT expression is consistent with previous studies indicating that transcription of *ctxAB* in vivo is preceded by the expression of TCP [104]. Late in infection, *Vibrios* down-regulate the expression of major virulence factors and detach to disseminate throughout the small intestine or return to the aquatic environment [173].

## Detachment

In the context of this article, we consider detachment the process by which cells in a sessile stage detach and switch to the planktonic (motile) lifestyle. The mechanism by which cells detach could include degradation of the substratum to which they are attached or cleavage of a protein or adhesin that anchors monolayers or multilayers of cells to a surface. In fully developed biofilm communities, detachment could be triggered by nutrient deprivation, accumulation of specific metabolites or toxic products, or as a consequence of external signals. The effective dispersion of motile *Vibrios* from a mature biofilm would require a certain degree of degradation of the biofilm matrix that holds cells together.

The soluble HA/protease was hypothesized to function as a "detachase" during infection based on the observation that *hapA* mutants remained attached for longer periods to Henle-407 cells [174], exhibited enhanced adherence to differentiated mucin-secreting HT29-18N2 cells [175], and elevated association with intestinal tissue compared to wild type [176]. HA/protease is a mucinase that is activated by quorum sensing and RpoS [125,177]. The "detachase activity" of HA/protease could partly result from its mucinase activity [178]. In addition, HA/protease was shown to degrade the GbpA adhesin required for attachment of *V*. *cholerae* to intestinal mucin [179]. A proteomic analysis showed that HA/protease is present in the matrix of biofilms formed in vitro [59]. HA/protease was recently shown to cleave RbmA, but this event increased biofilm formation rather than dispersal, as the RbmA cleavage product functioned to recruit planktonic cells to the growing biofilm [180].

**Fig 2:**
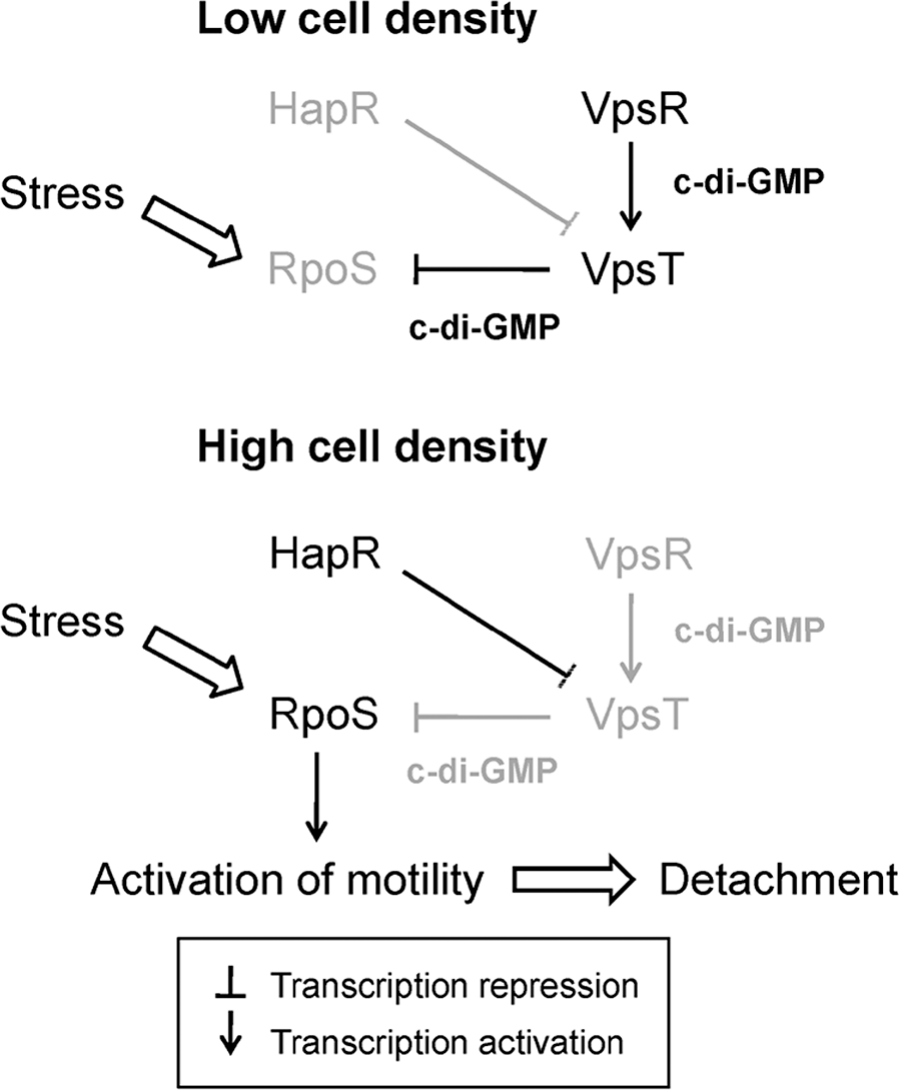
Model for quorum sensing and RpoS-dependent activation of motility and detachment. In a low cell density population, the regulator HapR is not expressed, the c-di-GMP content is high, and VpsT silences the transcription of *rpoS*. In a high cell density population, quorum sensing and stationary phase conditions induce the expression of HapR and RpoS that lower the c-di-GMP pool and terminate transcription of *vpsT*. In the absence of VpsT, RpoS is expressed to enhance motility. Activation of motility allows *Vibrios* to detach from a sessile community and swim away toward another unspent substratum. Inactive factors under each condition are indicated by a light grey font.

Reversal of a cell population from sessile to motile lifestyle is favored by environmentally induced downshifts in the c-di-GMP pool. Expression of HapR at high cell density and RpoS in stationary phase diminishes the c-di-GMP pool [37,122]. Lowering of the c-di-GMP pool enhances motility by increasing the activity of FlrA and diminishing the export of exopolysaccharides that stall the flagellum [49]. We have shown that transcription of *rpoN* encoding RNA polymerase σ^54^ subunit and *flrA* is directly diminished by H-NS and that expression of RpoS in the stationary phase counteracts this negative effect to enhance motility [181]. An RpoS-dependent activation of motility termed the "mucosal escape" response was first identified using the rabbit ileal loop model [182]. We have further shown that VpsT negatively impacts the mucosal escape response by repressing the transcription of *rpoS* [183]. In Fig 2, we unify these observations into a model for detachment involving quorum sensing, VpsT, and RpoS.

## Model for the Role of Biofilms in Intestinal Colonization and Pathogenesis

The genes required for the expression of flagellar motility and the biosynthesis of the biofilm exopolysaccharide and protein matrix (i.e., *vps*, *rbm*) are necessary for the efficient colonization of the small intestine. Therefore, the capacity of *V*. *cholerae* to adopt both lifestyles during infection could provide fitness in the environment of the gut. As shown in Table 1, flagellar motility could provide *Vibrios* with the advantage of mobility and capacity to spread along the gastrointestinal tract. On the other hand, biofilm formation could provide a mechanism of resistance to the host innate defense mechanism, facilitate a fast fecal–oral transmission route, and increase the fitness of those *Vibrios* that are directly shed back into the aquatic environment.

**Table 1:** Fitness benefits of *V*. *cholerae* dual lifestyle during intestinal colonization

Motile lifestyle	Sessile lifestyle
Motile *Vibrios* can swim toward the protective mucus coat and attach.	*Vibrio* biofilms exhibit enhanced infectivity.
Flagellar motility could contribute to the penetration of the protective mucus gel.	Biofilms could be more resistant to mechanical clearance.
The flagellum can cooperate with pili to facilitate *Vibrio* adherence to the protective mucus layer and underlying villi.	Biofilms could be more resistant to bile and antimicrobial peptides.
Flagellar motility participates in the regulation of virulence gene expression.	Biofilm formation could result in excretion of *Vibrios* in physiological stage more resistant to environmental stressors.

**Fig 3:**
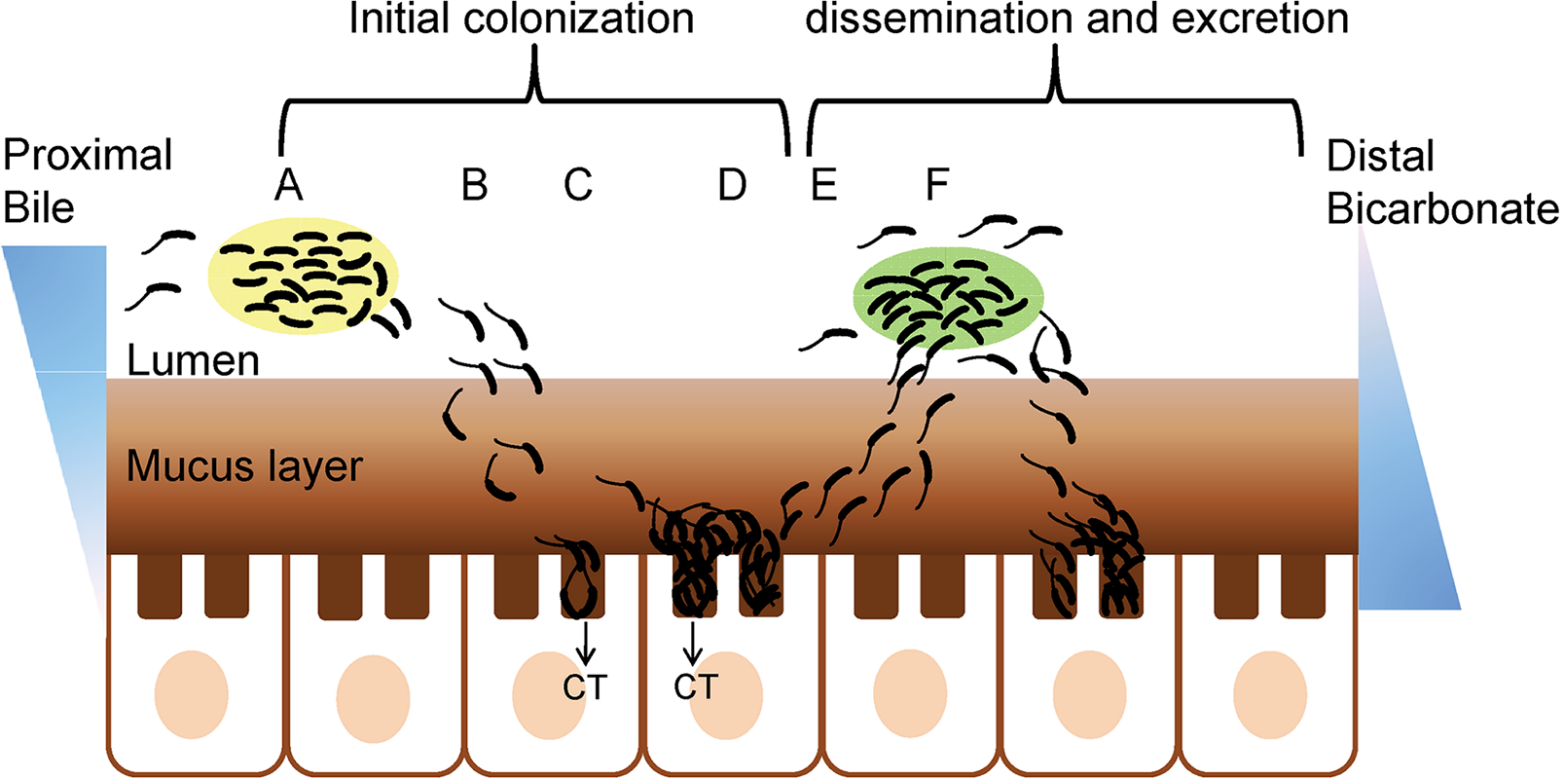
Model for the role of biofilm in intestinal colonization. (A) Cholera *Vibrios* can enter the small intestine as planktonic cells or embedded in a biofilm matrix, represented by a pale yellow shade. A fraction of *Vibrios* detach from the biofilm into the lumen. (B) Bile in the lumen acts as a repellent. *Vibrios* interact with the protective mucus coat and penetrate the mucus layer. (C) *Vibrios* interact with the villi. Bacterial interaction with the villi could involve iterative weak attachments that result in a more permanent adherence facilitated by TCP and other adhesins. Low bile, high bicarbonate, and cessation of motility in the proximity of the villi favor the expression of TCP and CT. (D) Expression of TCP and unknown factors promote microcolony formation along the villous axis and the crypts. (E) At high cell density, activation of HA/protease and motility by quorum sensing and RpoS promotes detachment. (F) Detached *Vibrios* are shed back into the luminal compartment. High bile concentration in the lumen enhances the c-di-GMP pool and favors biofilm formation. A fraction of detached *Vibrios* respond to bile stress by forming biofilms in vivo, indicated by *Vibrio* aggregates embedded in a pale green shade. Repetition of steps A through E spreads the infection along the small intestine. A mixture of *V*. *cholerae* planktonic cells, biofilm-like aggregates, and degraded mucus is excreted in the cholera stool.

In Fig 3, we provide a schematic view of the intestinal colonization process that takes into consideration the potential role of biofilm intake, their formation during infection, and excretion to the environment. *Vibrios* that enter the host in the form of a biofilm (Fig 3A) have a competitive advantage compared to planktonic cells. Planktonic cells detached from an infecting biofilm initially interact with the protective mucus barrier and move toward the underlying epithelium (Fig 3B). *Vibrios* are prevented from forming biofilm-like aggregates within the mucus gel by repression of *vps* genes (Fig 3B) [171]. *Vibrios* that fail to penetrate the protective mucus coat are passively excreted as a result of continuous mucus degradation and replenishment. The hallmark of intestinal colonization is adherence, multiplication, and microcolony formation along the villi and crypts (Fig 3C and 3D) [72]. Conditions in the villi (low bile, high bicarbonate) are less favorable for development of mature biofilms. Thus, we suggest that sessile microcolonies could represent cell aggregates remaining at an early stage of the biofilm development pathway. Late in infection, conditions of high cell density and nutrient limitation result in repression of virulence gene expression and detachment [173]. Detachment involves activation of motility and HA/protease by quorum sensing and RpoS [181–184]. This step, together with erosion of the mucus layer, re-exposes planktonic cells and/or microcolonies to the bactericidal effect of the elevated bile concentration present in the intestinal lumen (Fig 3E). Resistance to bile involves (i) the RND efflux pump [185], (ii) ToxR-dependent transcription activation of outer membrane protein OmpU [186], and (iii) ToxR activation of the LysR-family transcription activator LeuO [187], an activator of biofilm formation [34]. We suggest that detached planktonic cells or microcolonies could further aggregate into mature biofilms in the bile-rich luminal compartment (Fig 3F). Induction of *vps* and biofilm formation in the lumen could protect bacteria from bile killing [188], thereby allowing detached *Vibrios* to recolonize and disseminate along the small intestine (Fig 3). This interpretation explains the diminished intestinal colonization capacity of *vps* mutants [76]. Finally, *Vibrios* that exit the host in the form of a hyperinfective biofilm could have a higher probability of direct transmission to a secondary host (Fig 3F).

## Shortcomings and Caveats

A significant amount of work on the regulation of virulence gene expression and biofilm development has been concentrated in a relatively small number of strains of serogroup O1 (classical and El Tor biotype) and O139. The focus on a reduced number of strains favors the conception of molecular models but fails to represent the broad phenotypic and genetic diversity that occurs within serogroups and biotypes. Complex phenotypes such as virulence and biofilm development integrate numerous environmental cues and can exhibit strain-specific behavior, often resulting in conflicting data. Hence, the documented regulatory connections between virulence and biofilm expression summarized above should be appreciated in the context of genetic landscapes and environment conditions that can alter the expression of mutant phenotypes. An example of genetic diversity affecting virulence and biofilm formation is quorum sensing. In this case, some O1 lineages use the quorum sensing regulator HapR, and others employ the VieA regulatory system to respond to changes in cell density [189].

We also note that our limited understanding of the nature of sessile *V*. *cholerae* communities formed during infection comes with the caveat that biofilms formed under flow conditions in the small intestine could be structurally different from static biofilms formed in LB medium [190]. A variation of RIVET named recombination-based in-biofilm expression technology (RIBET) identified differences in the regulation of hydrodynamic versus static biofilms [191]. Interestingly, this study identified genes expressed in hydrodynamic biofilms that were also expressed late in infection [191].

In summary, though there is plentiful evidence for the formation of biofilms (or biofilm-like aggregates) during infection, the structure and regulation of these sessile communities remain largely unexplored.

**Key Learning Points**

■ *V*. *cholerae* requires the ability to alternate between motile and biofilm lifestyles to efficiently colonize the small intestine.
■ Motility and chemotaxis allow *V*. *cholerae* to move toward its niche in the small intestine and attach.
■ *V*. *cholerae* can form biofilm-like structures during infection.
■ *V*. *cholerae* biofilms are more resistant to stressful conditions in the host and exhibit a lower infective dose.
■ Biofilm formation and dispersal during infection could enhance dissemination of *V*. *cholerae* along the small intestine and its rapid transmission to a secondary host through a fecal–oral route.

**Top Five Papers**

1. Faruque SM, Biswas K, Udden SM, Ahmad QS, Sack DA, Nair GB, et al. Transmissibility of cholera: *in vivo*-formed biofilms and their relationship to infectivity and persistence in the environment. Proceedings of the National Academy of Sciences of the United States of America. 2006;103(16):6350–5.
2. Schild S, Tamayo R, Nelson EJ, Qadri F, Calderwood SB, Camilli A. Genes induced late in infection increase fitness of *Vibrio cholerae* after release into the environment. Cell host & microbe. 2007;2(4):264–77.
3. Tamayo R, Patimalla B, Camilli A. Growth in a biofilm induces a hyperinfectious phenotype in *Vibrio cholerae*. Infection and immunity. 2010;78(8):3560–9.
4. Fong JC, Syed KA, Klose KE, Yildiz FH. Role of *Vibrio* polysaccharide (*vps*) genes in VPS production, biofilm formation and *Vibrio cholerae* pathogenesis. Microbiology. 2010;156(Pt 9):2757–69.
5. Millet YA, Alvarez D, Ringgaard S, von Andrian UH, Davis BM, Waldor MK. Insights into *Vibrio cholerae* intestinal colonization from monitoring fluorescently labeled bacteria. PLoS Pathog. 2014;10(10):e1004405.
